# Punctuated Loci on Chromosome IV Determine Natural Variation in Orsay Virus Susceptibility of *Caenorhabditis elegans* Strains Bristol N2 and Hawaiian CB4856

**DOI:** 10.1128/JVI.02430-20

**Published:** 2021-05-24

**Authors:** Mark G. Sterken, Lisa van Sluijs, Yiru A. Wang, Wannisa Ritmahan, Mitra L. Gultom, Joost A. G. Riksen, Rita J. M. Volkers, L. Basten Snoek, Gorben P. Pijlman, Jan E. Kammenga

**Affiliations:** a Laboratory of Nematology, Wageningen University, Wageningen, The Netherlands; b Laboratory of Virology, Wageningen University, Wageningen, The Netherlands; c Theoretical Biology and Bioinformatics, Utrecht University, Utrecht, The Netherlands; University of Texas Southwestern Medical Center

**Keywords:** *Caenorhabditis elegans*, Orsay virus, QTL

## Abstract

Host-pathogen interactions play a major role in evolutionary selection and shape natural genetic variation. The genetically distinct Caenorhabditis elegans strains, Bristol N2 and Hawaiian CB4856, are differentially susceptible to the Orsay virus (OrV). Here, we report the dissection of the genetic architecture of susceptibility to OrV infection. We compare OrV infection in the relatively resistant wild-type CB4856 strain to the more susceptible canonical N2 strain. To gain insight into the genetic architecture of viral susceptibility, 52 fully sequenced recombinant inbred lines (CB4856 × N2 RILs) were exposed to OrV. This led to the identification of two loci on chromosome IV associated with OrV resistance. To verify the two loci and gain additional insight into the genetic architecture controlling virus infection, introgression lines (ILs) that together cover chromosome IV, were exposed to OrV. Of the 27 ILs used, 17 had an CB4856 introgression in an N2 background, and 10 had an N2 introgression in a CB4856 background. Infection of the ILs confirmed and fine-mapped the locus underlying variation in OrV susceptibility, and we found that a single nucleotide polymorphism in *cul-6* may contribute to the difference in OrV susceptibility between N2 and CB4856. An allele swap experiment showed the strain CB4856 became as susceptible as the N2 strain by having an N2 *cul-6* allele, although having the CB4856 *cul-6* allele did not increase resistance in N2. In addition, we found that multiple strains with nonoverlapping introgressions showed a distinct infection phenotype from the parental strain, indicating that there are punctuated locations on chromosome IV determining OrV susceptibility. Thus, our findings reveal the genetic complexity of OrV susceptibility in C. elegans and suggest that viral susceptibility is governed by multiple genes.

**IMPORTANCE** Genetic variation determines the viral susceptibility of hosts. Yet, pinpointing which genetic variants determine viral susceptibility remains challenging. Here, we have exploited the genetic tractability of the model organism Caenorhabditis elegans to dissect the genetic architecture of Orsay virus infection. Our results provide novel insight into natural determinants of Orsay virus infection.

## INTRODUCTION

Genetic variation plays a major role in the arms race between pathogen and host (1–3). The interaction between host genetic background and pathogen can shape natural variation by imposing a strong selection regime on the affected population. Host genetic variation plays a role in ongoing viral outbreaks as illustrated by studies that correlate outcome of infection with hepatitis virus, HIV, Zika virus, Ebola virus, and SARS-CoV-2 to the host’s genetic background (4–9). Studying host-virus interactions in model systems can uncover genetic networks determining viral susceptibility (10).

The nematode Caenorhabditis elegans encounters a variety of pathogens in its natural habitat, including bacteria, microsporidia, oomycetes, and fungi (11). Thus far, only one virus has been discovered that naturally infects C. elegans: the Orsay virus (OrV) (12). In the laboratory this pathogen can be easily maintained and used to study host-virus interactions (12). Host-virus interaction studies focusing on the effect of host genetic variation are facilitated by the androdiecious mode of reproduction by which C. elegans reproduces. This makes C. elegans a powerful model to investigate the effect of host genetic variation since populations can be both inbred and outcrossed.

Three cellular pathways are used by C. elegans to defend itself against viral infections. First, the RNA interference (RNAi) response is a highly adaptive and diverse pathway that plays a role in many processes in an organism, for example, in development and antiviral responses in invertebrates (13, 14). In OrV infection, it recognizes the double-stranded RNA replication intermediate, which ultimately leads to the production of small interfering RNAs (siRNAs) that target the viral RNA for degradation (15–19). Mutants defective for various genes in the RNAi pathway display higher viral susceptibility upon infection (15, 17–19). Second, the OrV can be targeted by a distinct mechanism known as viral uridylation (20). Uridylation, like RNAi, leads to degradation of viral RNAs, although both antiviral defenses function independently of one another. Third, the intracellular pathogen response (IPR) is involved in defense against viral, fungal, and microsporidian infections. The IPR is regulated by the gene pair *pals-22* and *pals-25* that balance the nematode’s physiological programs between growth and immunity (21). Infections are counteracted by upregulating a range of 80 IPR genes that reduce proteotoxic stress (21–23). For most IPR genes, their biochemical function is currently unknown, but IPR gene *cul-6* functions in the E3 ubiquitin ligase complex and protects against viral and microsporidian infection (23, 24). Furthermore, the gene *drh-1* (encoding a RIG-I like protein) mediates the IPR response specifically upon OrV infection connecting IPR and RNAi pathways which both depend on this gene (25).

Natural variation influences the susceptibility to OrV infections. Initially, it was observed that the natural C. elegans strain JU1580 is more susceptible to infection with OrV than the reference strain Bristol N2 (18). This difference has been linked to a natural polymorphism in *drh-1* affecting the antiviral RNAi response (15). In addition to the natural variation in the RNAi response, genetic variation also determines the IPR against OrV infection. The Hawaiian strain CB4856 had higher (basal) expression of multiple IPR genes than N2, potentially resulting in higher resistance to OrV infection observed in CB4856 (26). However, the genetic and transcriptional networks leading to this difference have not been uncovered.

The CB4856 and N2 strain are very polymorphic, with more than 400,000 polymorphisms, including insertions/deletions and single nucleotide variants (27, 28). Over the last decade, both strains have been jointly used in many quantitative genetics studies in C. elegans, focused on traits such as aging, stress tolerance, and pathogen avoidance (29–33). Most of these studies have been conducted on one of the two available recombinant inbred line (RIL) panels (34, 35) or on the introgression line (IL) population which contains fragments of CB4856 in a background of N2 (30).

Here, we set out to investigate genetic loci involved in the phenotypic differences between the Bristol N2 strain and the Hawaii CB4856 in response to OrV infection. Viral replication was characterized in N2 and CB4856 in a stage- and incubation time-dependent manner. Subsequently, we used inbred panels constructed from these strains to identify possible causal loci underlying the difference in viral susceptibility. We exposed a panel of 52 RILs to OrV and measured the viral load. We identified two quantitative trait loci (QTL) associated with differences in viral load on chromosome IV. Following-up, using a panel of 27 IL strains together covering the QTL location on chromosome IV led to the identification of 34 candidate genes involved in antiviral immunity. One of these candidate genes, the IPR gene *cul-6*, was tested for its role in OrV infection in the strains N2 and CB4856.

## RESULTS

### CB4856 displays resistance to OrV infection.

**FIG 1:**
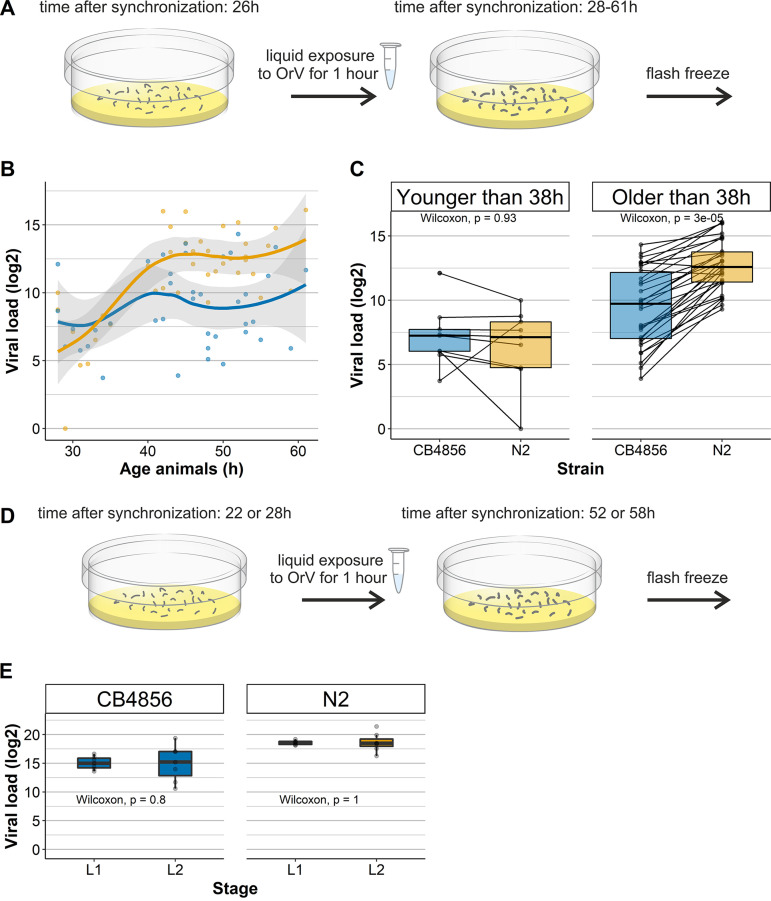
Kinetics of OrV infection in N2 and CB4856. (A) Nematodes are infected by the OrV in liquid at the age of 26 h (as in reference 17) before samples were washed from the plate 2 to 35 h later and collected for viral load quantification. (B) Development of OrV infection over time in N2 (orange) and CB4856 (blue) over a course of 35 h. The points depict the observed viral loads, the line represents the smoothed conditional mean, and the gray shading shows the confidence interval for the mean. (C) Box plot of viral load measurements over time for N2 and CB4856. The two panels show a division into two groups: early infection (up to 12 h postinfection) and late infection (after 12 h postinfection, green). Lines connecting the dots represent samples of the two genotypes measured in the same replicate at the same time point. (D) Viral infections either started in the L1 stage (22 h) or L2 stage (28 h). Populations were exposed to the OrV in liquid and isolated 30 h postinfection for viral load measurements. (E) Viral loads observed for N2 and CB4856 that were infected in the L1 or L2 stage.

The infection kinetics of OrV were investigated in the two wild-type strains N2 and CB4856. Infection kinetics were investigated by infecting both strains at an age of 26 h (L2 stage) and measuring the viral load over 2 to 35 h postinfection (in 28- to 61-h-old animals) (Fig. 1A). N2 developed a higher maximum viral load than CB4856 in this time period (Fig. 1B). The infection developed via a clear lag phase in N2 during the first 12 h, whereas large variation in viral loads was observed in the initial infection phase for CB4856 (Fig. 1B). In this time series experiment, a significant amount of the variance was explained by the different genotypes (permutational multivariate analysis of variance [PERMANOVA], *P* = 0.002). We found that for some infected CB4856 populations the infection developed via a similar pattern (but to lower viral load) compared to N2; however, in other experiments the infection did not develop beyond levels reached in the lag phase of the infection. Consequently, CB4856 populations that were 38 h or older showed either similar viral loads to populations that were younger (and thus shorter infected) or viral loads that reached the maximum viral load for CB4856 (Fig. 1C). On the other hand, N2 populations all reached higher viral loads after the lag-phase of infection was passed (Wilcoxon rank sum test, *P* < 1 × 10^−4^ Fig. 1C). Therefore, the time passed since infection also explained variation in viral load (PERMANOVA, *P* < 1 × 10^−4^). Next to this, we observed that infection was not always successfully established in both N2 (76% success rate) than and in CB4856 (61% success rate) populations (chi-square test, *P* = 0.093). Constant exposure to OrV for 4 days resulted in similar viral loads between N2 and CB4856 (15, 26), thus suggesting that multiple rounds of viral replication are necessary to fully infect CB4856 populations. Together, these observations show that CB4856 develops a lower viral load and can suppress a beginning infection better than N2.

A reason for the difference in viral load between CB4856 and N2 could be a stage-dependent difference in resistance as was found for CB4856 nematodes, which are resistant to infection by the microsporidian Nematocida parisii, but only in the L1 stage (36). Moreover, *N. parisii* shares its cellular tropism with OrV and both pathogens induce the same transcriptional response: the intracellular pathogen response (IPR) (21, 22, 37). Therefore, we also tested whether L1 CB4856 could exhibit even higher resistance to OrV infection than the L2 animals we have infected before. Infection was compared in first (22-h-old)- and second (28-h-old)-larval-stage animals (Fig. 1D). N2 animals were infected in parallel for reference and the infection could develop for 30 h after infection. We found for both genotypes that the viral loads were highly comparable between L1- and L2-infected nematodes (Wilcoxon rank sum test, *P* ≥ 0.8; Fig. 1E). Thus, the relative resistance of CB4856 toward the OrV is not stage dependent, in contrast to resistance to the microsporidian *N. parisii*.

### A locus on chromosome IV links to resistance against OrV.

**FIG 2:**
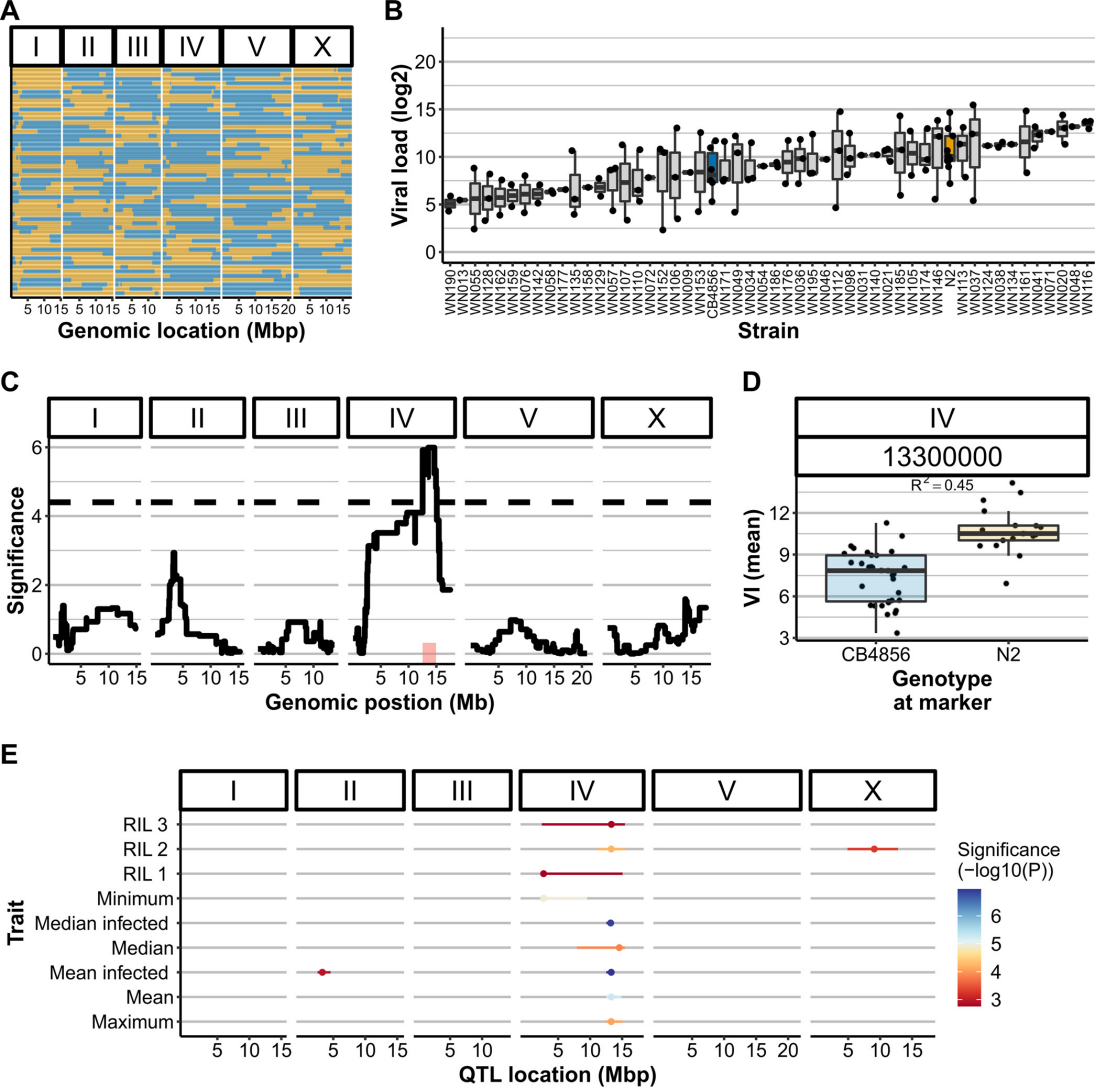
OrV infections in a RIL panel with parental strains N2 (orange) and CB4856 (blue) and QTL mapping. (A) Genetic map of the N2xCB4856 RIL panel. (B) Transgression plot of the viral loads of 52 RIL strains used for the infection assays Each dot represents a biological replicate of a viral load measured after infection. All RIL strains were infected three times, but samples that lacked viral replication (viral load = 0) are not shown. (C) QTL profile for mean viral load (excluding unsuccessful infections). The confidence interval is indicated in red, and a QTL peak is found at the end of chromosome IV at 13.3 Mb. (D) Split-out of the genotypes at the QTL peak, explaining 0.45 of the trait variation (Pearson correlation). (E) Overview of QTL patterns with other statistical summaries. The dots denote the QTL peak and the lines indicate the confidence interval. Note that the overall threshold is –log_10_(p) = 4.4; hence, not all peaks shown here are significant. However, most link to the right side of chromosome IV.

To find the causal genetic loci underlying the different viral loads between N2 and CB4856 in viral load, recombinant inbred lines (RILs) constructed from a cross between these strains were infected with OrV (Fig. 2A) (27, 35). The RILs were infected in the L2 stage (at the age of 26 h), and the infection was continued for 30 h, after which the viral load was measured. The viral loads of the RILs followed a pattern of transgressive segregation, indicating that multiple genetic loci contribute to viral susceptibility (Fig. 2B). We found a narrow-sense heritability (*h^2^*, the fraction of trait variation explained by genotype) of 0.56 for the mean viral load (excluding populations that were not successfully infected) meaning that 56% of this phenotype can be explained by additive genetic variance. Linkage analysis for this trait identified a QTL on chromosome IV between 12.5 and 14.9 Mb (Fig. 2C), linked to a higher viral load associated with N2 loci (Fig. 2D). Besides performing a linkage analysis for the mean viral load of successfully infected populations, linkage analysis was performed for (i) the mean and median viral load (both including and excluding unsuccessfully infected populations), (ii) the maximum and minimum viral load observed for a strain, and (iii) each RIL batch separately (Fig. 2E). Most of these summary statistics pointed toward the locus on the right-side of chromosome IV, but the minimum viral load pointed toward an additional QTL location with a peak at 2.7 Mb on chromosome IV (*R*^2^ = 0.32; Fig. 2E). Thus, this QTL location could link to the success of infection, whereas the QTL location on the right side of chromosome IV was linked to the height of the viral load measured. Therefore, each locus may influence another biological aspect of OrV infection.

### Verification of the QTL locus by introgression lines.

**FIG 3:**
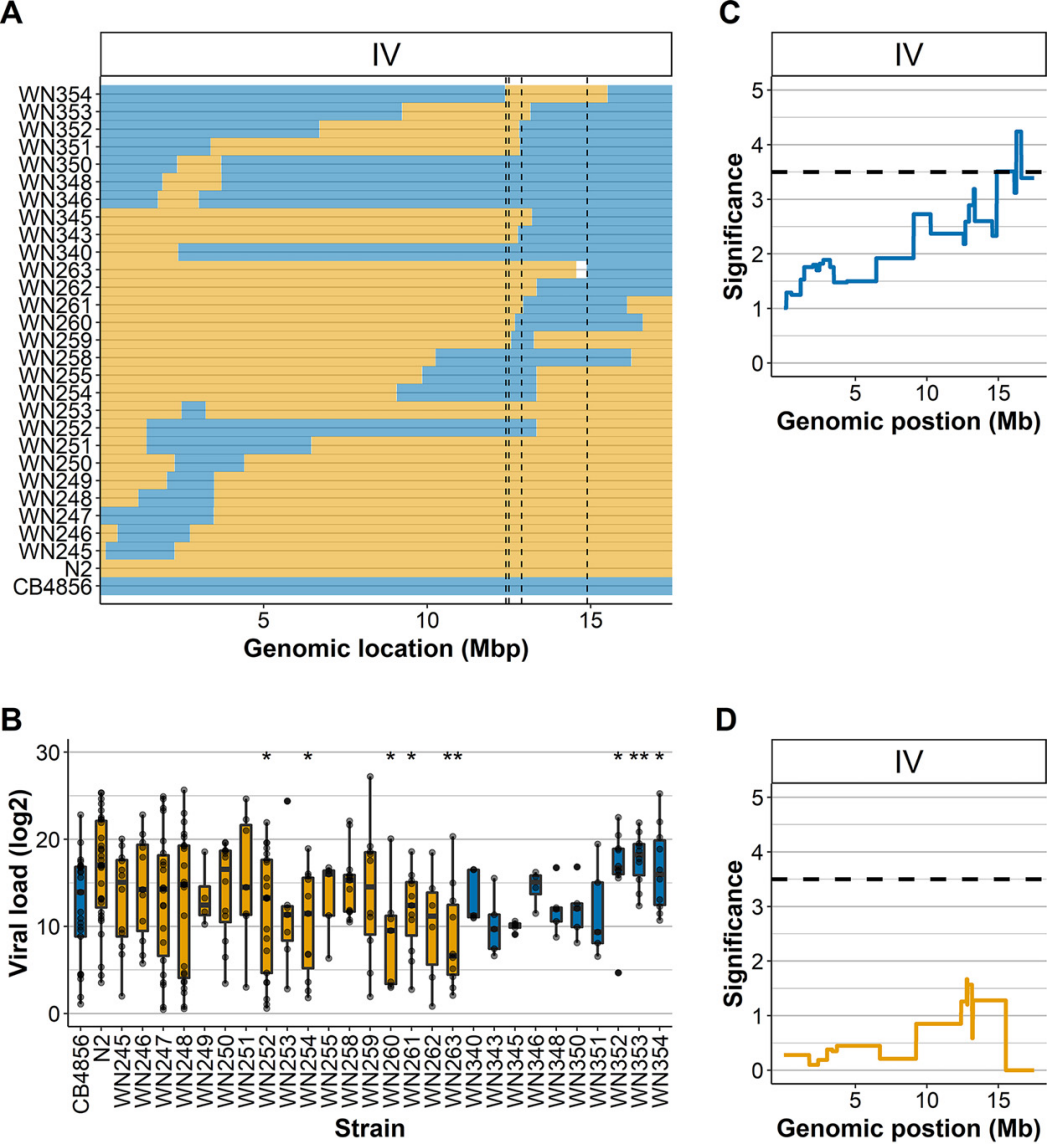
OrV infections in two IL panels with parental strains N2 and CB4856 and QTL mapping. (A) Genetic map of chromosome IV for the CB4856 background (strain WN340-WN354) and N2 background (WN245-WN263) ILs. The outer dashed lines indicate the QTL confidence interval as mapped in the RIL population, and the inner dashed lines indicate the area of interest identified in the IL_N2_ lines. (B) The viral loads of N2, CB4856, and 27 IL strains used for the infection assays. Of these, 17 strains have a CB4856 introgression in a N2 background (orange filled boxplots), and 10 have a N2 introgression in a CB4856 background (blue filled boxplots). An asterisk indicates a significant difference from its parental genetic background (*, *P* < 0.05; **, *P* < 0.01 [two-sided *t* test]). Each dot represents a biological replicate. Samples that lacked viral replication (viral load = 0) are not shown. (C) Linkage mapping profile for mean viral load (excluding unsuccessful infections) for the IL_N2_ panel, measuring the contribution of a CB4856 locus. A significant peak is found on the right side of chromosome IV. (D) Linkage mapping profile for mean viral load (excluding unsuccessful infections) for the IL_CB4856_ panel, measuring the contribution of an N2 locus.

To experimentally verify the QTL involved in the viral susceptibility difference between N2 and CB4856, ILs were infected. ILs contain small fragments of one strain in the genetic background of another strain (30). ILs that together cover chromosome IV were used and their viral loads were measured after infection. We used 10 ILs with a N2 fragment in the CB4856 background (IL_CB4856_) and 17 ILs with a CB4856 fragment in the N2 background (IL_N2_; Fig. 3A). Of the 27 infected ILs, 9 had a different viral load than the parental strain, demonstrating that presence of the introgression alters the viral susceptibility compared to the parent. We tested for recapitulation of the QTL effect (a higher viral load from an N2 allele and a lower viral load from a CB4856 allele). We found that the IL_CB4856_ strains WN352, WN353, and WN354 showed a phenotype distinct from the parental CB4856 strain (two-sided *t* test, *P* < 0.05; Fig. 3B). These strains carry introgressions that together overlap the right QTL peak at 12.41 to 12.89 Mb (Fig. 3A). In agreement, four IL_N2_ strains covering this QTL were more resistant than N2 (WN252, WN254, WN260, and WN261) (Fig. 3A and B). In contrast, three strains with the CB4856 fragment in the N2 background covering the same location did not show a lower viral load than the N2 strain (WN255, WN258, and WN259). Furthermore, two IL_CB4856_ strains fully covering this introgression (WN345 and WN351) did also not confirm the locus. In addition, IL_N2_ strain WN263 with an introgression from 14.87 to 17.49 Mb had a lower susceptibility than N2. These results indicate that there are multiple loci underlying the susceptibility difference between N2 and CB4856 that are likely to interact together.

Linkage analysis on the IL_N2_ panel showed the highest correlations for mean viral load and genetic background on the right side of chromosome IV with a QTL peak at around 16 Mb (Fig. 3C), whereas the IL_CB4856_ panel mapping did not show an effect of the introgression (Fig. 3D). The resolution for QTL mapping in the ILs is relatively low compared to QTL mapping in the RILs, because of fewer genetic breakpoints in the population. Therefore, the peak mapped in the ILs could rely on the same genetic variation as the QTL peak mapping in the RIL panel that estimated a QTL between 12.5 and 14.9 Mb.

### Candidate causal genes underlying different viral susceptibility between N2 and CB4856.

**FIG 4:**
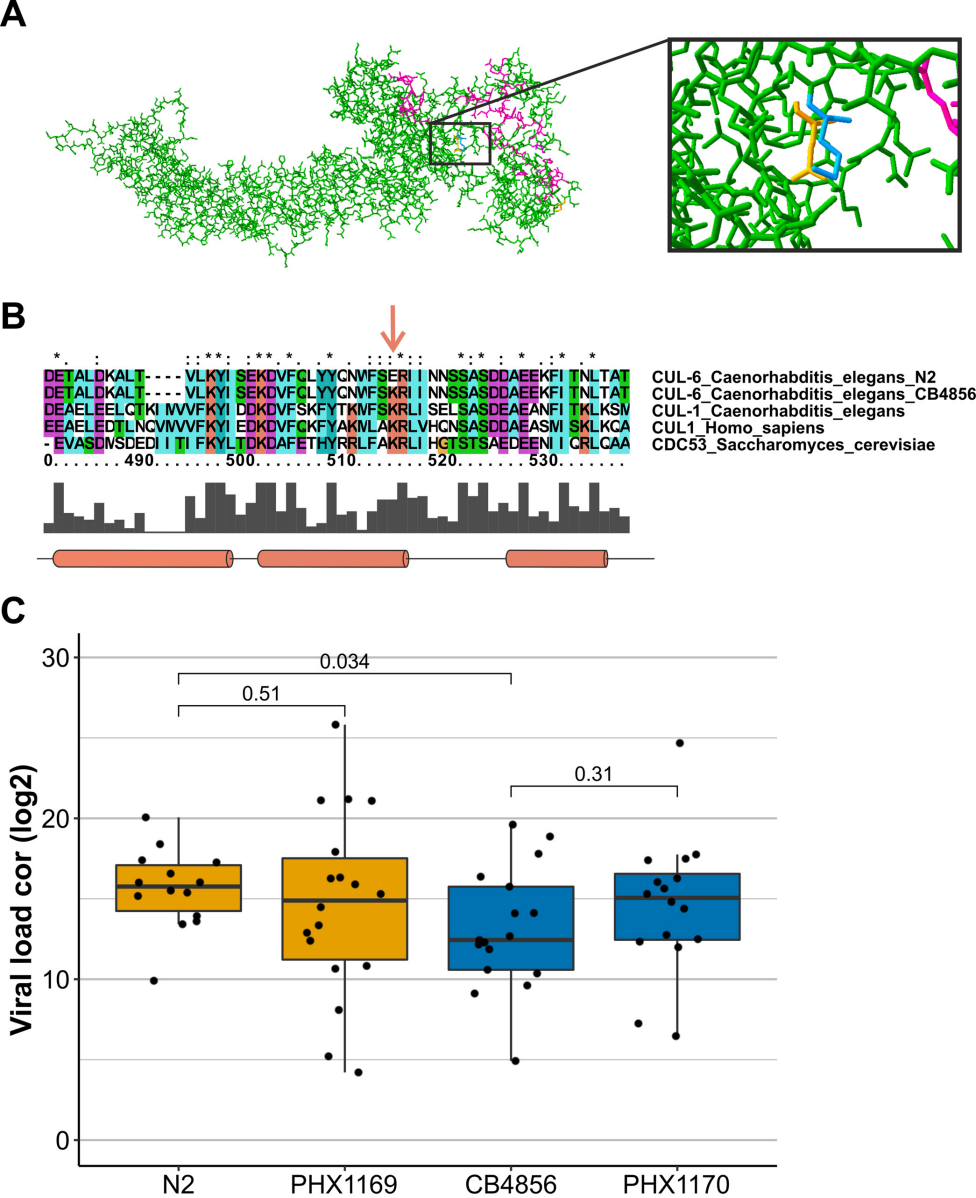
*cul-6* gene in CB4856 and N2 and its effect on viral susceptibility. (A) Structure prediction of C. elegans CUL-6. The lysine present in the CB4856 allelic variant is shown is blue, and the glutamic acid present in the N2 allelic variant in orange. The RBX-1 binding domain is shown in purple. (B) Part of the sequence alignment between Homo sapiens CUL1, Saccharomyces cerevisiae CDC53, C. elegans CUL-1, and the C. elegans N2 and CB4856 allelic variants of CUL-6. The location of the N2 and CB4856 polymorphism is indicated with an arrow. The amino acid conservation is indicated by the gray bars at the bottom and by the annotations on top (single dot, weakly conserved; double dot, strongly conserved; asterisk, completely conserved). The gene *cul-6* contains a polymorphism between N2 and CB4856 at a conserved site. Colors are based on the amino acid properties, and locations of alpha-helices are indicated by cylinders (38). (C) Viral susceptibility of N2, CB4856, PHX1169 (N2 genetic background carrying a CB4856 *cul-6* allele), and PHX1170 (CB4856 genetic background carrying a N2 allele) (significances from a *t* test).

Linkage analysis in both RILs and ILs indicated that viral susceptibility differences between N2 and CB4856 were governed by multiple loci. So, we set out to determine whether we could identify polymorphic genes that determine the difference in viral susceptibility. We focus on the 12.41- to 12.89-Mb region on chromosome IV, because this region was mapped in the RIL panel and supported by analysis of the ILs. This region contains 34 polymorphic genes, 25 of which contain a nonsynonymous change in the coding sequence (see Table S3 in the supplemental material). The candidate genes in this region have diverse functions, including genes with a known immune function against bacterial or viral infection. One of these is *cul-6*, which is regulated by the IPR. A knockdown of *cul-6* increases the susceptibility to OrV in N2 nematodes (21, 22, 24). The CB4856 allele of *cul-6* gene contains a single nucleotide polymorphism in amino acid 428, physically close to the RBX-1 binding site, where a negatively charged glutamic acid is found in N2 and a positively charged lysine in CB4856 (Fig. 4A). The amino acid lysine at this position has been highly conserved from yeast to humans in the closely related CDC53 and CUL-1 proteins (amino acid conservation between C. elegans CUL-1 and CUL-6 is 47%) (Fig. 4B) (38).

To test whether the *cul-6* polymorphism explains the difference in viral susceptibility between N2 and CB4856, we used CRISPR-Cas9-modified strains encoding the *cul-6* N2 allele in the CB4856 genetic background (PHX1170) and the *cul-6* CB4856 allele in the N2 genetic background (PHX1169). Based on the results of the IL analysis, PHX1170 was expected to be as susceptible as N2. We indeed observed that PHX1170 had a more susceptible phenotype, with a viral load in between that of N2 (*P* = 0.41) and CB4856 (*P* = 0.31) (see Fig. S3 and Table S4 in the supplemental material). PHX1169 retained high viral susceptibility with more variance in observed viral loads than in the N2 strain. These findings, taken together, show that the *cul-6* polymorphism may contribute to different viral susceptibility between N2 and CB4856, and yet the effect size of this allele is modest. The resistant phenotype of CB4856 cannot be fully allocated to this allele because it does not confer resistance in a susceptible background and CB4856 and PHX1170 were more similar than CB4856 and N2. Taken together with the results from the ILs, these results suggest that having the susceptible *cul-6* allele from N2 makes the strains vulnerable to infection while having the resistant allele from CB4856 does not protect strains with an otherwise susceptible N2 background.

## DISCUSSION

Here, we have unraveled the genetic architecture of viral susceptibility in the C. elegans strains N2 and CB4856. We found two QTL peaks on chromosome IV linking to susceptibility differences and confirmed the QTL on the right side of chromosome IV using a selection of ILs. Observations made for individual ILs show that multiple loci on chromosome IV contribute to viral susceptibility. When we zoomed in on the 12.4- to 12.9-Mb region that likely contains a causal gene, we identified 34 polymorphic genes which may explain differences in viral susceptibility between N2 and CB4856. Allele swap experiments between one of these candidate genes, the IPR gene *cul-6*, indicated a single nucleotide polymorphism may underlie susceptibility differences. Furthermore, we found that other genetic loci on chromosome IV also contribute to the whole phenotypic variation between N2 and CB4856. These findings show that the genetic architecture of OrV susceptibility is a complex, polygenic trait, and future studies may identify even more genetic variants involved in OrV susceptibility.

### Chromosome IV is implicated in natural variation in OrV infection.

By exposing RILs and ILs to OrV, we identified a QTL on chromosome IV that is implicated in a lower viral load due to the CB4856 allele. A genome-wide association study (GWAS) on OrV infection in C. elegans also involved chromosome IV (15), but in contrast to that study, we did not find a QTL near the *drh-1* locus. This was in line with expectations since only two polymorphisms are found in the introns between N2 and CB4856 for this gene (27). Still, the more distal associations uncovered by the GWAS could potentially result from the same allelic variation as the QTL between 12.41 and 12.89 Mb because the GWAS identified five locations on chromosome IV which are between 5 and 13 Mb. Therefore, natural populations of C. elegans may carry similar genetic variants conferring OrV resistance as N2 and CB4856.

In our previous study investigating viral susceptibility differences between N2 and CB4856 we found that CB4856 had higher basal expression of IPR genes which we hypothesized may be caused by distinctive *pals-22*/*pals-25* expression patterns (26). These genes, the respective repressor and activator of the IPR, are located adjacent to each other on the left of chromosome III (21). eQTL studies showed local genetic variation (*cis*-eQTL) regulates expression of *pals-22* and *pals-25* (17, 29, 35, 39–42). Nevertheless, we did not observe a link between natural genetic variation in viral susceptibility in N2 and CB4856 and the *pals-22*/*pals-25* locus on chromosome III. Our results show that we could only explain a minor fraction of the heritability by the QTL locations we found. This result is typical for QTL mappings of complex traits and suggests that additional loci contribute to the viral susceptibility difference between N2 and CB4856. These loci may have small effect sizes, interactions or are affected by a (currently unknown) environmental cause (43).

Interestingly, the higher resistance of CB4856 to both the microsporidium *N. parisii* and the OrV appears to be governed by distinct mechanisms, despite both types of infections are counteracted by the IPR (21, 22, 37). Balla et al. have previously found that CB4856 nematodes displayed higher pathogen resistance than N2 when infected with the microsporidium *N. parisii*. However, this higher resistance was only observed when L1 nematodes were infected (36). Exposure to *N. parisii* in the L2, L3, and L4 stages yielded similar pathogen loads between N2 and CB4856 animals (36). In contrast, we showed here that both L1- and L2-exposed CB4856 nematodes convey higher resistance to the OrV than N2 nematodes. In line with these results, the genetic basis of resistance to OrV and *N. parisii* can be found on distinct chromosomes. Here, we attributed OrV resistance to chromosome IV, whereas QTL mapping identified chromosome II, III, and V contributed to resistance against *N. parisii* (36). The observations further indicate that anti-stress responses in C. elegans can be highly specialized, despite commonalities between them (23, 25).

### Orsay virus susceptibility has a polygenic basis.

The QTL in the RIL panel and follow-up fine-mapping in the ILs identified a relatively small locus that contributes to the viral susceptibility toward OrV infection. We investigated the effect of a *cul-6* polymorphism and found that this SNP could contribute to viral susceptibility. This allele appears to function in one direction by making the resistant CB4856 background susceptible when carrying the N2-allele, despite the N2 *cul-6* allele also has an antiviral effect as demonstrated by in RNAi knockdown experiments (24). Thus, the CB4856 allele could either have a stronger antiviral function than the N2 *cul-6* allele or the latter does not function as well in a CB4856 background. Notably, both allele versions are commonly present in natural C. elegans populations (44).

However, the phenotypic difference between N2 and CB4856 cannot be entirely explained by the *cul-6* allele alone. The 12.4- to 12.9-Mb region also contains other genes that may affect viral susceptibility. Some of these are transcriptionally activated by OrV infection, others have more general or unknown cellular functions (16, 19, 22, 24, 37, 45). Besides, the 12.4- to 12.9-Mb region specifically investigated here, we show that are multiple other loci and genes on chromosome IV contribute to viral susceptibility. The left side of chromosome IV appeared to be involved in determining the success of infection, but we could not verify this result in all the ILs. Studying this QTL may be complicated because only a small fraction of infections fails. Nevertheless, some ILs covering the left side of chromosome IV had a viral susceptibility distinct from the parent. In addition, strain WN351 carries a susceptible introgression at the 12.4- to 12.9-Mb locus but remained resistant. This strain has a large introgression also covering the left side of chromosome IV, where interacting genes may be located. Furthermore, we have some indication (not significant) that the left-side of chromosome II affects viral load. This is noteworthy because genetic variation between N2 and CB4856 in this region contributes to microsporidian resistance (36). Therefore, it may be worthwhile infecting ILs with introgressions on chromosome II to further investigate its potential role in OrV susceptibility.

Our results reveal part of the complex genetic basis of OrV susceptibility. These results are in line with other studies mapping variation in viral susceptibility to the hosts’ genome (see, for example, references 4 and 46–49). Because viruses use the host’s cellular machinery to replicate and hosts have multiple mechanisms to counteract viruses, host-virus interactions will comprise many genetic interactions that can be affected by genetic variation. Thus, future studies may aim to uncover genetic networks rather than a single gene to further enhance our understanding of natural variation in host-virus interactions.

## MATERIALS AND METHODS

### *C. elegans* strains.

C. elegans strains Bristol N2 and Hawaiian CB4856 were used and strains derived by crossing these two wild-type strains. Here, 52 RILs, 17 ILs with an N2-background (IL_N2_) and 10 ILs with a CB4856 background (IL_CB4856_) covering chromosome IV were used. IL strains and RIL strains are described in Table S1 in the supplemental material. The RILs were described previously (35). All these genotypes have been confirmed by whole-genome sequencing on the Illumina HiSeq 2500 platform as described by previously (50).

The strains PHX1169 *cul-6*(*syb1169*) and PHX1170 *cul-6*(*syb1170*), containing the *cul-6* CB4856 allele in a N2 background and the N2 *cul-6* allele in a CB4856 background, respectively, have been created by CRISPR-Cas9 by SunyBiotech (see File S1 in the supplemental material). These genotypes have been confirmed by PCR sequencing. For *syb1169* the primers SL01-Seq-s (AAGTGTTGTCTCTGAGTTGC) and SL01-Seq-a (CGGATTAAGAGATCCTACGA) were used, and for *syb1170* the primers SL02-SEQ-S (AAGTGTTGTCTCTGAGTTGC) and SL02-SEQ-A (CGGATTAAGAGATCCTACGA) were used for sequencing.

### *C. elegans* culturing.

The nematodes were kept at 12°C in-between experiments on 6-cm nematode growth medium (NGM) plates seeded with Escherichia coli OP50. Bleaching was used to synchronize populations and to remove bacterial or fungal contaminations (51). Before experiments, a population without males was created by picking single worms in the L1/L2 stage and transferring hermaphrodite populations to fresh 9-cm NGM plates. New experiments were started by bleaching an egg-laying population grown at 20°C.

### Orsay virus stock preparation.

Orsay virus stocks were generated by isolating OrV from a persistently infected JU1580 culture as previously described (17, 18). In short, JU1580 populations were grown on 100 9-cm NGM plates (51) containing twice the usual amount of agar to prevent the nematodes from burying into the agar (34 g/liter). The nematodes were collected by washing the animals off the plate with M9 buffer (51) and flash freezing the suspension in liquid nitrogen. After defrosting on ice, the supernatant was collected and passed through a 0.2-μm filter. Specific infectivity of the virus stock was tested by serial dilution infections in C. elegans JU1580 (17). Depending on the infectivity, 50 μl or 100 μl OrV/500 μl infection solution was used.

### Infection experiments.

The infection assay was conducted as described previously (17). In short, populations were synchronized (*t* = 0 h) by bleaching and grown at 20°C on 9-cm NGM plates. Per plate, approximately 300 animals were grown. Infections were performed on animals in the L1 (22 h postbleaching) or L2 (26 or 28 h postbleaching as indicated in the text) stage. Prior to the infection, the nematodes were washed off the plate with M9 buffer and pelleted by centrifugation. The supernatant was removed, and the nematodes were exposed to OrV in liquid for 1 h. For the L1/L2 comparison, RIL and IL experiments, and replicates 8 to 21 of the allele-swap experiment, stock titers determined at 50 μl/500 μl were used. For the replication kinetics and replicates 1 to 7 of the allele-swap experiment, stock titers determined at 100 μl/500 μl were used. The worms were washed three times with M9 and placed on a fresh 9-cm NGM plate.

For the replication kinetics experiments on N2 and CB4856, the animals were harvested 2 to 35 h postinfection. The replication kinetics experiment was conducted eight times, and for each of these biological replicates the animals were harvested at four to five different time points per replicate, equally covering the time series for N2 and CB4856. Together, these eight experiments cover at least two independent biological replicates per strain for every hour in the 2- to 35-h time course except for 35 to 39 h and 60 h postinfection.

For the viral load experiments on the L1/L2 comparison, RIL and IL panels and the *cul-6* allele swap strains, the animals were harvested 30 h postinfection. The experiment in the RIL panel was conducted on three independent biological replicates. The experiment in the IL panel was conducted on at least five independent biological replicates. The experiment comparing L1/L2 nematodes was conducted on five independent biological replicates. The experiment using the *cul-6* allele swap strains was conducted on 21 independent biological replicates.

### RNA isolation.

The RNA was isolated using a Maxwell 16 AS2000 instrument with a Maxwell 16 LEV simply RNA tissue kit (both Promega) according to the recommended protocol, except for the addition of 10 mg of proteinase K during the lysis step. The lysate was incubated in a Thermomixer (Eppendorf) for 10 min at 65ºC at 1,000 rpm. After isolation the quality and quantity of the RNA was determined via NanoDrop (Thermo Scientific).

### cDNA preparation and qPCR.

cDNA was synthesized using the GoScript reverse transcriptase kit (Promega) according to the recommended protocol with random hexanucleotides (Thermo Scientific) and 1 μg of total RNA as starting material. The cDNA was quantified by qPCR (MyIQ; Bio-Rad) using Absolute QPCR SYBR green fluorescein mixes (Thermo Scientific) or iQ SYBR green Supermix (Bio-Rad) according to the recommended protocol. The samples were quantified using the primers described by (17).

The qPCR data were processed using R (version 4.0.3), as described previously (17). In short, before normalization, the qPCR measurements were transformed as determined by the following equation:

$$Q_{\mathrm{gene}}=2^{40-CT_{\text{gene}}}$$

where *Q*_gene_ is the expression of the gene and *CT_gene_* is the measured threshold cycle (*C_T_*) value of the gene. The viral expression was normalized by the two reference genes, using the formula:

$$E=\frac{Q_{V}}{0.5*\left(\left(Q_{\mathrm{rp}16}/\bar{Q_{\mathrm{rp}16}}\right)+\left(Q_{Y37E3.8}/\bar{Q_{Y37E3.8}}\right)\right)}$$

where *E* is the normalized viral load, *Q_V_* is the expression of the viral RNA and *Q_rp_*_16_ and *Q*_Y37E3.8_ are the expression of reference genes *rpl-6* and Y37E3.8, respectively. Viral load data presented here was batch corrected for the batch effect caused by the different virus stock used by correcting for the average viral loads of N2 and CB4856 (excluding unsuccessful infections) as these two strains were taken along in every experiment.

From the replicate measurements in the RIL panel, several traits could be derived for QTL mapping over the RIL population. The following parameters were derived, including all measurements: mean viral load, median viral load, and minimum viral load. We excluded the unsuccessful infections (as these could arise due to technical failures) unless indicated otherwise.

### Data analysis.

Quantitative data were analyzed in R (version 4.0.3). We used the “vegan” and “heritability” packages for specific analyses as indicated below (52, 53). Furthermore, for data organization and plotting the “tidyverse” packages were used (54).

### Analysis of the infection kinetics differences between N2 and CB4856.

For testing the differences in infection kinetics between N2 and CB4856, PERMANOVA was used from the vegan package (the *adonis* function) (52). We ran an interaction model only distinguishing between pre- and post-log-phase *E* = Strain ⋅ Age_pre/post_ with 10,000 permutations.

To test differences in successful infections, a chi-squared test was performed on the number of successful infections using the *chisq.test* function. For this, we used all L2 infection observations made in the experiments presented here (115 infections in CB4856 and 121 infections in N2).

### Quantitative trait locus mapping RIL population.

Single locus QTL mapping was done using a linear model to explain viral load and derived traits over the markers by

$$E_{i}∼x_{i,j}+\;\varepsilon_{i,j}$$

where *E* is the viral load of RIL *i* (1, 2, …, 52) and *x* is the marker of RIL *i* at location *j* (a set of 1,152 sequenced markers was used [see Table S2 in the supplemental material]) (27). For *E*, the outcome of each replicate of the experiment was averaged over the three biological replicates. The QTL confidence interval was determined by a drop of 2 in the log_10_(p) value [e.g., if the peak was –log_10_(p) = 6, then the boundary of the QTL was at –log_10_(p) = 4.0].

For the QTL mapping, the statistical threshold was determined via a permutation analysis, where the values measured for *E* were randomly distributed over the genotypes. The same model as for the mapping was used and this analysis was repeated 1,000 times. The 950th highest *P* value was taken as the *P* value threshold for a false discovery rate of 0.05.

The variance explained by the QTL peak was calculated using the *cor* function (with Pearson correlation), correlating the trait variation with the genotype at the peak-marker.

### Heritability and variance calculations.

The narrow-sense heritability’s (*h^2^*) were calculated per investigated trait by REML (14, 55, 56) using the “heritability” package (53). Significance was determined via 1,000 permutations, where the values measured for *E* were randomly distributed over the genotypes.

The variation of viral loads explained by a QTL peak (*V*_Explained_) was calculated by:

$$V_{\mathrm{Explained}}=\frac{R_{\mathrm{QTL}}^{2}}{h^{2}}$$

where *R^2^_QTL_* is the determination coefficient from fitting the peak marker to the trait as calculated by a linear model and *h^2^* is the narrow-sense heritability of the trait.

### Introgression line analysis.

The viral loads obtained for the introgression lines were analyzed individually against N2 and CB4856 via a two-sided *t* test assuming unequal variance. Experiments where no virus was detected were excluded from the analysis. Moreover, we performed linkage mapping for the two IL panels separately using a linear model to explain viral load over the markers by

$$E_{i}∼x_{i,j}+\;\varepsilon_{i,j}$$

where *E* is the viral load of IL *i* (1, 2, …, 10 or 17) and *x* is the marker of IL *i* at location *j* (a set of 1,152 sequenced markers was used [see Table S2]). Each IL was compared against the respective parental strain (N2 or CB4856). For *E* the outcome of each replicate of the experiment was averaged over the biological replicates. A significance threshold was drawn at –log_10_(p) > 3.5 for analysis of the data.

### Allele swap analysis.

Because we observed a high level of variance in the viral loads in N2 and CB4856 and the effect size of the QTL_IV:12.41-12.89_ was small, we used a high level of replication for the allele swap experiments by performing 21 biologically independent infections using three different virus stocks. Unsuccessful infections were excluded from the analysis and the batch corrected viral load data (based on virus stock as described above) was subsequently checked for outliers. Outliers were defined by 1.5 times the interquartile range plus or minus the third or first quartile, respectively. After removal of the outliers (7% of the measurements), a *t* test assuming unequal variances was performed to test for differences in viral load.

### Protein structure analysis.

Protein sequences from the human CUL1 (NCBI reference sequence NP_003583.2), Saccharomyces cerevisiae CDC53 (GenBank accession no. CAA98702.1), Drosophila melanogaster CUL-1 (GenBank AAD33676.1) and C. elegans CUL-1 (GenBank AAC47120.1), CUL-6 N2 allelic variant (GenBank CAB01230.1), and CUL-6 CB4856 allelic variant were aligned using ClustalX (version 2.1) with the default settings (57). A structural model for the N2 and CB4856 allelic variant was predicted using the human CUL1 protein structure as a template in the SWISS-MODEL ExPASy web server. The default search parameters were used, based on the SWISS-MODEL template library (version 14/01/2015) and the protein data bank (version 09/01/2015) (58–63). The obtained models for N2 and CB4856 CUL-6 were compared in SwissPDBViewer (v4.1.0) (64).

### Data availability.

All scripts and underlying data are available at https://git.wur.nl/published_papers/sterken_sluijs_2020. In addition, the QTL experiment will be included in the next version of WormQTL to facilitate interactive use of the data (32).

## References

[1] Obbard DJ, Jiggins FM, Halligan DL, Little TJ. 2006. Natural selection drives extremely rapid evolution in antiviral RNAi genes. Curr Biol 16:580–585. 10.1016/j.cub.2006.01.065. 16546082

[2] Vasseur E, Patin E, Laval G, Pajon S, Fornarino S, Crouau-Roy B, Quintana-Murci L. 2011. The selective footprints of viral pressures at the human RIG-I-like receptor family. Hum Mol Genet 20:4462–4474. 10.1093/hmg/ddr377. 21865300

[3] Enard D, Cai L, Gwennap C, Petrov DA. 2016. Viruses are a dominant driver of protein adaptation in mammals. Elife 5:e12469. 10.7554/eLife.12469. 27187613 PMC4869911

[4] Heim MH, Bochud P-Y, George J. 2016. Host-hepatitis C viral interactions: the role of genetics. J Hepatol 65:S22–S32. 10.1016/j.jhep.2016.07.037. 27641986

[5] Rasmussen AL, Okumura A, Ferris MT, Green R, Feldmann F, Kelly SM, Scott DP, Safronetz D, Haddock E, LaCasse R, Thomas MJ, Sova P, Carter VS, Weiss JM, Miller DR, Shaw GD, Korth MJ, Heise MT, Baric RS, de Villena FP-M, Feldmann H, Katze MG. 2014. Host genetic diversity enables Ebola hemorrhagic fever pathogenesis and resistance. Science 346:987–991. 10.1126/science.1259595. 25359852 PMC4241145

[6] Dean M, Carrington M, Winkler C, Huttley GA, Smith MW, Allikmets R, Goedert JJ, Buchbinder SP, Vittinghoff E, Gomperts E, Donfield S, Vlahov D, Kaslow R, Saah A, Rinaldo C, Detels R, O’Brien SJ. 1996. Genetic restriction of HIV-1 infection and progression to AIDS by a deletion allele of the CKR5 structural gene. Science 273:1856–1862. 10.1126/science.273.5283.1856. 8791590

[7] Yun S, Song B, Frank JC, Julander JG, Olsen AL, Polejaeva IA, Davies CJ, White KL, Lee Y. 2018. Functional genomics and immunologic tools : the impact of viral and host genetic variations on the outcome of Zika virus infection. Viruses 10:422–428. 10.3390/v10080422. PMC6116225 30103523

[8] Nguyen A, David JK, Maden SK, Wood MA, Weeder BR, Nellore A, Thompson RF. 2020. Human leukocyte antigen susceptibility map for severe acute respiratory syndrome coronavirus 2. J Virol 94:1–12. 10.1128/JVI.00510-20. PMC7307149 32303592

[9] Hou Y, Zhao J, Martin W, Kallianpur A, Chung MK, Jehi L, Sharifi N, Erzurum S, Eng C, Cheng F. 2020. New insights into genetic susceptibility of COVID-19: an ACE2 and TMPRSS2 polymorphism analysis. BMC Med 18:1–8. 10.1186/s12916-020-01673-z. 32664879 PMC7360473

[10] van Sluijs L, Pijlman GP, Kammenga JE. 2017. Why do individuals differ in viral susceptibility? A story told by model organisms. Viruses 9:284–213. 10.3390/v9100284. PMC5691635 28973976

[11] Schulenburg H, Félix M. 2017. The natural biotic environment of *Caenorhabditis elegans*. Genetics 206:55–86. 10.1534/genetics.116.195511. 28476862 PMC5419493

[12] Félix M-A, Wang D. 2019. Natural viruses of *Caenorhabditis* nematodes. Annu Rev Genet 53:4.1–4.14. 10.1146/annurev-genet-112618-043756 31424970

[13] Tabara H, Sarkissian M, Kelly WG, Fleenor J, Grishok A, Timmons L, Fire A, Mello CC. 1999. The *rde-1* gene, RNA interference, and transposon silencing in *Caenorhabditis elegans*. Cell 99:123–132. 10.1016/S0092-8674(00)81644-X. 10535731

[14] Grishok A, Mello C. 2002. RNAi (nematodes: *Caenorhabditis elegans*), p 339–360. *In* Homology effects. Elsevier, New York, NY. 10.1016/s0065-2660(02)46012-9 11931230

[15] Ashe A, Bélicard T, Le Pen J, Sarkies P, Frézal L, Lehrbach NJ, Félix M-A, Miska EA. 2013. A deletion polymorphism in the *Caenorhabditis elegans* RIG-I homolog disables viral RNA dicing and antiviral immunity. Elife 2:e00994. 10.7554/eLife.00994. 24137537 PMC3793227

[16] Sarkies P, Ashe A, Le Pen J, McKie MA, Miska EA. 2013. Competition between virus-derived and endogenous small RNAs regulates gene expression in *Caenorhabditis elegans*. Genome Res 23:1258–1270. 10.1101/gr.153296.112. 23811144 PMC3730100

[17] Sterken MG, Snoek LB, Bosman KJ, Daamen J, Riksen JAG, Bakker J, Pijlman GP, Kammenga JE. 2014. A heritable antiviral RNAi response limits Orsay virus infection in *Caenorhabditis elegans* N2. PLoS One 9:e89760. 10.1371/journal.pone.0089760. 24587016 PMC3933659

[18] Félix MA, Ashe A, Piffaretti J, Wu G, Nuez I, Bélicard T, Jiang Y, Zhao G, Franz CJ, Goldstein LD, Sanroman M, Miska EA, Wang D. 2011. Natural and experimental infection of *Caenorhabditis* nematodes by novel viruses related to nodaviruses. PLoS Biol 9:e1000586. 10.1371/journal.pbio.1000586. 21283608 PMC3026760

[19] Tanguy M, Véron L, Stempor P, Ahringer J, Sarkies P, Miska EA. 2017. An alternative STAT signaling pathway acts in viral immunity in *Caenorhabditis elegans*. mBio 8:e00924-17. 10.1128/mBio.00924-17. 28874466 PMC5587905

[20] Le Pen J, Jiang H, Di Domenico T, Kneuss E, Kosałka J, Leung C, Morgan M, Much C, Rudolph KLM, Enright AJ, O’Carroll D, Wang D, Miska EA. 2018. Terminal uridylyltransferases target RNA viruses as part of the innate immune system. Nat Struct Mol Biol 25:778–786. 10.1038/s41594-018-0106-9. 30104661 PMC6130846

[21] Reddy KC, Dror T, Underwood RS, Osman GA, Elder CR, Desjardins CA, Cuomo CA, Barkoulas M, Troemel ER. 2019. Antagonistic paralogs control a switch between growth and pathogen resistance in *Caenorhabditis elegans*. PLoS Pathog 15:e1007528. 10.1371/journal.ppat.1007528. 30640956 PMC6347328

[22] Reddy KC, Dror T, Sowa JN, Panek J, Chen K, Lim ES, Wang D, Troemel ER. 2017. An intracellular pathogen response pathway promotes proteostasis in *Caenorhabditis elegans*. Curr Biol 27:3544–3553.e5. 10.1016/j.cub.2017.10.009. 29103937 PMC5698132

[23] Panek J, Gang SS, Reddy KC, Luallen RJ, Fulzele A, Bennett EJ, Troemel ER. 2020. A cullin-RING ubiquitin ligase promotes thermotolerance as part of the intracellular pathogen response in *Caenorhabditis elegans*. Proc Natl Acad Sci U S A 117:7950–7960. 10.1073/pnas.1918417117. 32193347 PMC7148578

[24] Bakowski MA, Desjardins CA, Smelkinson MG, Dunbar TL, Dunbar TA, Lopez-Moyado IF, Rifkin SA, Cuomo CA, Troemel ER. 2014. Ubiquitin-mediated response to microsporidia and virus infection in *Caenorhabditis elegans*. PLoS Pathog 10:e1004200. 10.1371/journal.ppat.1004200. 24945527 PMC4063957

[25] Sowa JN, Jiang H, Somasundaram L, Tecle E, Xu G, Wang D, Troemel ER. 2019. The *Caenorhabditis elegans* RIG-I homolog DRH-1 mediates the intracellular pathogen response upon viral infection. J Virol 94:e01173-19. 10.1128/JVI.01173-19. PMC6955277 31619561

[26] van Sluijs L, Bosman K, Pankok F, Blokhina T, Riksen JAG, Snoek BL, Pijlman GP, Kammenga JE, Sterken MG. 2019. Balancing selection shapes the intracellular pathogen response in natural *Caenorhabditis elegans* populations. bioRxiv https://www.biorxiv.org/content/10.1101/579151v5. 10.3389/fcimb.2021.758331 PMC8841876 35174100

[27] Thompson OA, Snoek LB, Nijveen H, Sterken MG, Volkers RJMM, Brenchley R, van’t Hof A, Bevers RPJJ, Cossins AR, Yanai I, Hajnal A, Schmid T, Perkins JD, Spencer D, Kruglyak L, Andersen EC, Moerman DG, Hillier LWDW, Kammenga JE, Waterston RH, Edgley M, Strasbourger P, Flibotte S, Ewing B, Adair R, Au V, Chaudhry I, Fernando L, Hutter H, Kieffer A, Lau J, Lee N, Miller A, Raymant G, Shen B, Shendure J, Taylor J, Turner EH, Moerman DG, Waterston RH, Snoek LB, Nijveen H, Sterken MG. 2015. Remarkably divergent regions punctuate the genome assembly of the *Caenorhabditis elegans* Hawaiian strain CB4856. Genetics 200:975–989. 10.1534/genetics.115.175950. 25995208 PMC4512556

[28] Kim C, Kim J, Kim S, Cook DE, Evans KS, Andersen EC, Lee J. 2019. Long-read sequencing reveals intra-species tolerance of substantial structural variations and new subtelomere formation in *Caenorhabditis elegans*. Genome Res 29:1023–1035. 10.1101/gr.246082.118. 31123081 PMC6581047

[29] Viñuela A, Snoek LB, Riksen JAG, Kammenga JE. 2012. Aging uncouples heritability and expression-QTL in *Caenorhabditis elegans*. G3 (Bethesda) 2:597–605. 10.1534/g3.112.002212. 22670229 PMC3362942

[30] Doroszuk A, Snoek LB, Fradin E, Riksen J, Kammenga J. 2009. A genome-wide library of CB4856/N2 introgression lines of CB4856/N2 introgression lines of *Caenorhabditis elegans*. Nucleic Acids Res 37:e110. 10.1093/nar/gkp528. 19542186 PMC2760803

[31] Rodriguez M, Snoek LB, Riksen JAG, Bevers RP, Kammenga JE. 2012. Genetic variation for stress-response hormesis in *Caenorhabditis elegans* lifespan. Exp Gerontol 47:581–587. 10.1016/j.exger.2012.05.005. 22613270

[32] Snoek BL, Sterken MG, Hartanto M, van Zuilichem AJ, Kammenga JE, de Ridder D, Nijveen H. 2020. WormQTL2: an interactive platform for systems genetics in *Caenorhabditis elegans*. Database 2020:1–17. 10.1093/database/baz149. 31960906 PMC6971878

[33] Nakad R, Snoek LB, Yang W, Ellendt S, Schneider F, Mohr TG, Rösingh L, Masche AC, Rosenstiel PC, Dierking K, Kammenga JE, Schulenburg H. 2016. Contrasting invertebrate immune defense behaviors caused by a single gene, the *Caenorhabditis elegans* neuropeptide receptor gene *npr-1*. BMC Genomics 17:280. 10.1186/s12864-016-2603-8. 27066825 PMC4827197

[34] Rockman MV, Kruglyak L. 2009. Recombinational landscape and population genomics of *Caenorhabditis elegans*. PLoS Genet 5:e1000419. 10.1371/journal.pgen.1000419. 19283065 PMC2652117

[35] Li Y, Álvarez OA, Gutteling EW, Tijsterman M, Fu J, Riksen JAG, Hazendonk E, Prins P, Plasterk RHA, Jansen RC, Breitling R, Kammenga JE. 2006. Mapping determinants of gene expression plasticity by genetical genomics in *Caenorhabditis elegans*. PLoS Genet 2:e222–e261. 10.1371/journal.pgen.0020222. 17196041 PMC1756913

[36] Balla KM, Andersen EC, Kruglyak L, Troemel ER. 2015. A wild *Caenorhabditis elegans* strain has enhanced epithelial immunity to a natural microsporidian parasite. PLoS Pathog 11:e1004583. 10.1371/journal.ppat.1004583. 25680197 PMC4334554

[37] Chen K, Franz CJ, Jiang H, Jiang Y, Wang D. 2017. An evolutionarily conserved transcriptional response to viral infection in *Caenorhabditis* nematodes. BMC Genomics 18:303. 10.1186/s12864-017-3689-3. 28415971 PMC5392922

[38] Zheng N, Schulman BA, Song L, Miller JJ, Jeffrey PD, Wang P, Chu C, Koepp DM, Elledge SJ, Pagano M, Conaway RC, Conaway JW, Harper JW, Pavletich NP. 2002. Structure of the Cul1–Rbx1–Skp1–F boxSkp2 SCF ubiquitin ligase complex. Nature 416:703–709. 10.1038/416703a. 11961546

[39] Rockman MV, Skrovanek SS, Kruglyak L. 2010. Selection at linked sites shapes heritable phenotypic variation in *Caenorhabditis elegans*. Science 330:372–376. 10.1126/science.1194208. 20947766 PMC3138179

[40] Li Y, Breitling R, Snoek LB, Van Der Velde KJ, Swertz MA, Riksen J, Jansen RC, Kammenga JE. 2010. Global genetic robustness of the alternative splicing machinery in *Caenorhabditis elegans*. Genetics 186:405–410. 10.1534/genetics.110.119677. 20610403 PMC2940304

[41] Viñuela A, Snoek LB, Riksen JAG, Kammenga JE. 2010. Genome-wide geneexpression regulation as a function of genotype and age in *Caenorhabditis elegans*. Genome Res 20:929–937. 10.1101/gr.102160.109. 20488933 PMC2892094

[42] Snoek BL, Sterken MG, Bevers RPJ, Volkers RJM, van’t Hof A, Brenchley R, Riksen JAG, Cossins A, Kammenga JE. 2017. Contribution of trans-regulatory eQTL to cryptic genetic variation in *Caenorhabditis elegans*. BMC Genomics 18:1–15. 10.1186/s12864-017-3899-8. 28662696 PMC5492678

[43] Eichler EE, Flint J, Gibson G, Kong A, Leal SM, Moore JH, Nadeau JH. 2010. Missing heritability and strategies for finding the underlying causes of complex disease. Nat Rev Genet 11:446–450. 10.1038/nrg2809. 20479774 PMC2942068

[44] Cook DE, Zdraljevic S, Roberts JP, Andersen EC. 2017. CeNDR, the *Caenorhabditis elegans* natural diversity resource. Nucleic Acids Res 45:D650–D657. 10.1093/nar/gkw893. 27701074 PMC5210618

[45] Lee RYN, Howe KL, Harris TW, Arnaboldi V, Cain S, Chan J, Chen WJ, Davis P, Gao S, Grove C, Kishore R, Muller HM, Nakamura C, Nuin P, Paulini M, Raciti D, Rodgers F, Russell M, Schindelman G, Tuli MA, Van Auken K, Wang Q, Williams G, Wright A, Yook K, Berriman M, Kersey P, Schedl T, Stein L, Sternberg PW. 2018. WormBase 2017: molting into a new stage. Nucleic Acids Res 46:D869–D874. 10.1093/nar/gkx998. 29069413 PMC5753391

[46] van Manen D, van ‘T Wout AB, Schuitemaker H. 2012. Genome-wide association studies on HIV susceptibility, pathogenesis, and pharmacogenomics. Retrovirology 9:70. 10.1186/1742-4690-9-70. 22920050 PMC3468375

[47] Al-Qahtani A, Khalak HG, Alkuraya FS, Al-Hamoudi W, Al-Hamoudy W, Alswat K, Al Balwi MA, Al Abdulkareem I, Sanai FM, Abdo AA. 2013. Genome-wide association study of chronic hepatitis B virus infection reveals a novel candidate risk allele on 11q22.3. J Med Genet 50:725–732. 10.1136/jmedgenet-2013-101724. 24065354

[48] McLaren PJ, Coulonges C, Bartha I, Lenz TL, Deutsch AJ, Bashirova A, Buchbinder S, Carrington MN, Cossarizza A, Dalmau J, De Luca A, Goedert JJ, Gurdasani D, Haas DW, Herbeck JT, Johnson EO, Kirk GD, Lambotte O, Luo M, Mallal S, van Manen D, Martinez-Picado J, Meyer L, Miro JM, Mullins JI, Obel N, Poli G, Sandhu MS, Schuitemaker H, Shea PR, Theodorou I, Walker BD, Weintrob AC, Winkler CA, Wolinsky SM, Raychaudhuri S, Goldstein DB, Telenti A, de Bakker PIW, Zagury J-F, Fellay J. 2015. Polymorphisms of large effect explain the majority of the host genetic contribution to variation of HIV-1 virus load. Proc Natl Acad Sci U S A 112:14658–14663. 10.1073/pnas.1514867112. 26553974 PMC4664299

[49] Nedelko T, Kollmus H, Klawonn F, Spijker S, Lu L, Hessman M, Alberts R, Williams RW, Schughart K. 2012. Distinct gene loci control the host response to influenza H1N1 virus infection in a time-dependent manner. BMC Genomics 13:411. 10.1186/1471-2164-13-411. 22905720 PMC3479429

[50] Gao AW, Sterken MG, de Bos J, van Creij J, Kamble R, Snoek BL, Kammenga JE, Houtkooper RH. 2018. Natural genetic variation in *Caenorhabditis elegans* identified genomic loci controlling metabolite levels. Genome Res 28:1296–1308. 10.1101/gr.232322.117. 30108180 PMC6120624

[51] Brenner S. 1974. The genetics of *Caenorhabditis elegans*. Genetics 77:71–94. 10.1093/genetics/77.1.71. 4366476 PMC1213120

[52] Oksanen J, Blanchet FG, Friendly M, Kindt R, Legendre P, McGlinn D, Minchin PR, O’Hara RB, Simpson GL, Solymos P, Stevens MHH, Szoecs E, Wagner H. 2020. Vegan: community ecology package. https://cran.r-project.org/web/packages/vegan/index.html.

[53] Kruijer W, with a contribution from White I. 2019. Heritability: marker-based estimation of heritability using individual plant or plot data. (Contains data collected by Flood P, Kooke R.) https://cran.r-project.org/web/packages/heritability/index.html.

[54] Wickham H. 2019. Tidyverse: easily install and load the “Tidyverse.” https://cran.r-project.org/web/packages/tidyverse/index.html.

[55] Kruijer W, Boer MP, Malosetti M, Flood PJ, Engel B, Kooke R, Keurentjes JJB, Van Eeuwijk FA. 2015. Marker-based estimation of heritability in immortal populations. Genetics 199:379–398. 10.1534/genetics.114.167916. 25527288 PMC4317649

[56] Speed D, Hemani G, Johnson MR, Balding DJ. 2012. Improved heritability estimation from genome-wide SNPs. Am J Hum Genet 91:1011–1021. 10.1016/j.ajhg.2012.10.010. 23217325 PMC3516604

[57] Larkin MA, Blackshields G, Brown NP, Chenna R, Mcgettigan PA, McWilliam H, Valentin F, Wallace IM, Wilm A, Lopez R, Thompson JD, Gibson TJ, Higgins DG. 2007. Clustal W and Clustal X version 2.0. Bioinformatics 23:2947–2948. 10.1093/bioinformatics/btm404. 17846036

[58] Remmert M, Biegert A, Hauser A, Söding J. 2012. HHblits : lightning-fast iterative protein sequence searching by HMM-HMM alignment. Nat Methods 9:173–175. 10.1038/nmeth.1818. 22198341

[59] Altschul SF, Madden TL, Schäffer AA, Zhang J, Zhang Z, Miller W, Lipman DJ. 1997. Gapped BLAST and PSI-BLAST : a new generation of protein database search programs. Nucleic Acids Res 25:3389–3402. 10.1093/nar/25.17.3389. 9254694 PMC146917

[60] Guex N, Peitsch MC. 1997. SWISS-MODEL and the Swiss-PdbViewer : an environment for comparative protein modeling. Electrophoresis 18:2714–2723. 10.1002/elps.1150181505. 9504803

[61] Šali A, Blundell TL. 1993. Comparative protein modeling by satisfaction of spatial restraints. J Mol Biol 234:779–815. 10.1006/jmbi.1993.1626. 8254673

[62] Benkert P, Biasini M, Schwede T. 2011. Toward the estimation of the absolute quality of individual protein structure models. Bioinformatics 27:343–350. 10.1093/bioinformatics/btq662. 21134891 PMC3031035

[63] Mariani V, Kiefer F, Schmidt T, Haas J, Schwede T. 2011. Template based assessment of template based protein structure predictions in CASP9. Proteins 79:37–58. 10.1002/prot.23177. 22002823

[64] Guex N, Peitsch MC, Schwede T. 2009. Automated comparative protein structure modeling with SWISS-MODEL and Swiss-PdbViewer: a historical perspective. Electrophoresis 30:S162–S173. 10.1002/elps.200900140. 19517507

