# Supplementary material for: Punctuated Loci on Chromosome IV Determine Natural Variation in Orsay Virus Susceptibility of Caenorhabditis elegans Strains Bristol N2 and Hawaiian CB4856
Source: J Virol. 2021 May 24;95(12):e02430-20. doi: 10.1128/JVI.02430-20 (PMC8315983; doi:10.1128/JVI.02430-20)
Supplement: Supplementary file 4 [file jvi.02430-20-s0001.pdf]

Strain name and genotype：PHX1169 cul-6(syb1169)

>SL01-syb1169

aagtgttgtctctgagttgctcatccatactatgtatgtactgccgtacaacttgggatccctgaataatttttcggaaaa
ctactagaaacttccggaaagtttgttaaaactgtcagaaatgttgagcaaactttttttaaaagtttcagaaattttcaga
gttgttcaaaagtattgaattgaataccatttttagaaaaataaacattctaattctaaacaaacgaagtttcagGGTGTA
TCTCGAAAAGCGAATTTTAAAAGAAGGACACGAAACATTGCAAAGACTGGCTAAAGACAGTGGACTAAAAACTACACCAAA
GGAATACATAACCAAACTCCTTGAAGTTCACGAGATTTATTTCAACTTGATAAATAAAGCATTCGATAGAAATGCACTTTT
CATGCAATCTCTCGACAAAGCTTCTAAGGACTTTATCGAAGCTAATGCTGTGACTATGTTGGCTCCTGAAAAACACCGAAG
CACAAGGTCTGCAGACTATCTTGCAAGATATTGTGATCAGCTCTTGAAGAAGAATTCC**AAA**GTTCAAGATGAAACGGCGCT
AGATAAAGCAgtgagtttgctttttttcaactctgaacgtcgctgctccatcacatttttctgtacgaaaaattccctttat
agatcaaaacatgatgggacagcctaacaccacgtatgtaaaaaaatcccattttcag**CTG**ACGGTGCTCAAG**TAC**ATCAG
CGAAAAGGATGTCTTCCAATTGTATTATCAAAATTGGTTC**TCGAAGCGC**ATCATCAATAATTCTTCAGCAAGTGATGATGC
AGAAGAAAAGTTCATTACAAATCTAACTGCAACAGAAGGTCTCGAATATACTCGCAATCTTGTGAAAATGGTCGAAGACGC
TAAAATTAGTAAAGATTTGACTACTGAATTCAAAGATATCAAAACGGAAAAGTCGATTGACTTTAACGTTATTCTTCAAAC
AACTGGTGCATGGCCAAGTTTAGAT**CAG**ATCAAGATTATTCTTCCG**CGT**GAATTATCGACAATTCTCAAAGAGTTCGATAC
ATTCTACAATGCGAGTCATAATGGACGAAGATTGAACTGGGCTTACTCACAATGCCGGGGCGAAGTTAACTCTAAAGCTTT
CGAAAAGAAATATGTATTTATTgtgagtttatctatagttattcaaacttattatttttatcagataacattttattaaaa
attttaaaatcagaagtcagcctgaagcagaagcagaagaagaagccaaactgctttttgaaatcttaacttattaaaagt
tagaattttccagGTTACAGCAAGCCAACTCTGCACGCTCTATCTTTTCAATGAGCAGGACTCGTTCACAATTGAACAAAT
TTCCAAAGCTATTGAAATGACTGCAAAGTCAACTTCGGCTATCGTAGGATCTCTTAATCCG

>N2

aagtgttgtctctgagttgctcatccatactatgtatgtactgccgtacaacttgggatccctgaataatttttcggaaaa
ctactagaaacttccggaaagtttgttaaaactgtcagaaatgttgagcaaactttttttaaaagtttcagaaattttcaga
gttgttcaaaagtattgaattgaataccatttttagaaaaataaacattctaattctaaacaaacgaagtttcagGGTGTA
TCTCGAAAAGCGAATTTTAAAAGAAGGACACGAAACATTGCAAAGACTGGCTAAAGACAGTGGACTAAAAACTACACCAAA
GGAATACATAACCAAACTCCTTGAAGTTCACGAGATTTATTTCAACTTGATAAATAAAGCATTCGATAGAAATGCACTTTT
CATGCAATCTCTCGACAAAGCTTCTAAGGACTTTATCGAAGCTAATGCTGTGACTATGTTGGCTCCTGAAAAACACCGAAG
CACAAGGTCTGCAGACTATCTTGCAAGATATTGTGATCAGCTCTTGAAGAAGAATTCC**AAG**GTTCAAGATGAAACGGCGCT
AGATAAAGCAgtgagtttgctttttttcaactctgaacgtcgctgctccatcacatttttctgtacgaaaaattccctttat
agatcaaaacatgatgggacagcctaacaccacgtatgtaaaaaaatcccattttcag**TTA**ACGGTGCTCAAG**TAT**ATCAG
CGAAAAGGATGTCTTCCAATTGTATTATCAAAATTGGTTC**AGTGAACGA**ATCATCAATAATTCTTCAGCAAGTGATGATGC
AGAAGAAAAGTTCATTACAAATCTAACTGCAACAGAAGGTCTCGAATATACTCGCAATCTTGTGAAAATGGTCGAAGACGC
TAAAATTAGTAAAGATTTGACTACTGAATTCAAAGATATCAAAACGGAAAAGTCGATTGACTTTAACGTTATTCTTCAAAC
AACTGGTGCATGGCCAAGTTTAGAT**CAA**ATCAAGATTATTCTTCCG**CGA**GAATTATCGACAATTCTCAAAGAGTTCGATAC
ATTCTACAATGCGAGTCATAATGGACGAAGATTGAACTGGGCTTACTCACAATGCCGGGGCGAAGTTAACTCTAAAGCTTT
CGAAAAGAAATATGTATTTATTgtgagtttatctatagttattcaaacttattatttttatcagataacattttattaaaa
attttaaaatcagaagtcagcctgaagcagaagcagaagaagaagccaaactgctttttgaaatcttaacttattaaaagt
tagaattttccagGTTACAGCAAGCCAACTCTGCACGCTCTATCTTTTCAATGAGCAGGACTCGTTCACAATTGAACAAAT
TTCCAAAGCTATTGAAATGACTGCAAAGTCAACTTCGGCTATCGTAGGATCTCTTAATCCG

Strain name and genotype：PHX1170 cul-6(syb1170)

>SL02-syb1170

aagtgttgtctctgagttgctcatccatactatgtatgtactgccgtacaacttgggatccctgaataatttttcggaaaa
ctactagaaacttccggaaagtttgttaaaactgtcagaaatgttgagcaaactttttttaaaagtttcagaaattttcaga
gttgttcaaaagtattgaattgaataccatttttagaaaaataaacattctaattctaaacaaacgaagtttcagGGTGTA
TCTCGAAAAGCGAATTTTAAAAGAAGGACACGAAACATTGCAAAGACTGGCTAAAGACAGTGGACTAAAAACTACACCAAA
GGAATACATAACCAAACTCCTTGAAGTTCACGAGATTTATTTCAACTTGATAAATAAAGCATTCGATAGAAATGCACTTTT
CATGCAATCTCTCGACAAAGCTTCTAAGGACTTTATCGAAGCTAATGCTGTGACTATGTTGGCTCCTGAAAAACACCGAAG
CACAAGGTCTGCAGACTATCTTGCAAGATATTGTGATCAGCTCTTGAAGAAGAATTCC**AAA**GTTCAAGATGAAACGGCGCT
AGATAAAGCAgtgagtttgctttttttcaactctgaacgtcgctgctccatcacatttttctgtacgaaaaattccctttat
agatcaaaacatgatgggacagcctaacaccacgtatgtaaaaaaatcccatttttcag**CTG**ACGGTGCTCAAG**TAC**ATCAG
CGAAAAGGATGTCTTCCAATTGTATTATCAAAATTGGTTC**TCGGAGCGC**ATCATCAATAATTCTTCAGCAAGTGATGATGC
AGAAGAAAAGTTCATTACAAATCTAACTGCAACAGAAGGTCTCGAATATACTCGCAATCTTGTGAAAATGGTCGAAGACGC
TAAAATTAGTAAAGATTTGACTACTGAATTCAAAGATATCAAAACGGAAAAGTCGATTGACTTTAACGTTATTCTTCAAAC
AACTGGTGCATGGCCAAGTTTAGAT**CAG**ATCAAGATTATTCTTCCG**CGT**GAATTATCGACAATTCTCAAAGAGTTCGATAC
ATTCTACAATGCGAGTCATAATGGACGAAGATTGAACTGGGCTTACTCACAATGCCGGGGCGAAGTTAACTCTAAAGCTTT
CGAAAAGAAATATGTATTTATTgtgagtttatctatagttattcaaacttattatttttatcagataacattttattaaaa
attttaaaatcagaagtcagcctgaagcagaagcagaagaagaagccaaactgctttttgaaatcttaacttattaaaagt
tagaattttccagGTTACAGCAAGCCAACTCTGCACGCTCTATCTTTTCAATGAGCAGGACTCGTTCACAATTGAACAAAT
TTCCAAAGCTATTGAAATGACTGCAAAGTCAACTTCGGCTATCGTAGGATCTCTTAATCCG

>CB4856

aagtgttgtctctgagttgctcatccatactatgtatgtactgccgtacaacttgggatccctgaataatttttcggaaaa
ctactagaaacttccggaaagtttgttaaaactgtcagaaatgttgagcaaactttttttaaaagtttcagaaattttcaga
gttgttcaaaagtattgaattgaataccatttttagaaaaataaacattctaattctaaacaaacgaagtttcagGGTGTA
TCTCGAAAAGCGAATTTTAAAAGAAGGACACGAAACATTGCAAAGACTGGCTAAAGACAGTGGACTAAAAACTACACCAAA
GGAATACATAACCAAACTCCTTGAAGTTCACGAGATTTATTTCAACTTGATAAATAAAGCATTCGATAGAAATGCACTTTT
CATGCAATCTCTCGACAAAGCTTCTAAGGACTTTATCGAAGCTAATGCTGTGACTATGTTGGCTCCTGAAAAACACCGAAG
CACAAGGTCTGCAGACTATCTTGCAAGATATTGTGATCAGCTCTTGAAGAAGAATTCC**AAG**GTTCAAGATGAAACGGCGCT
AGATAAAGCAgtgagtttgctttttttcaactctgaacgtcgctgctccatcacatttttctgtacgaaaaattccctttat
agatcaaaacatgatgggacagcctaacaccacgtatgtaaaaaaatcccatttttcag**TTA**ACGGTGCTCAAG**TAT**ATCAG
CGAAAAGGATGTCTTCCAATTGTATTATCAAAATTGGTTC**AGTAAACGA**ATCATCAATAATTCTTCAGCAAGTGATGATGC
AGAAGAAAAGTTCATTACAAATCTAACTGCAACAGAAGGTCTCGAATATACTCGCAATCTTGTGAAAATGGTCGAAGACGC
TAAAATTAGTAAAGATTTGACTACTGAATTCAAAGATATCAAAACGGAAAAGTCGATTGACTTTAACGTTATTCTTCAAAC
AACTGGTGCATGGCCAAGTTTAGAT**CAA**ATCAAGATTATTCTTCCG**CGA**GAATTATCGACAATTCTCAAAGAGTTCGATAC
ATTCTACAATGCGAGTCATAATGGACGAAGATTGAACTGGGCTTACTCACAATGCCGGGGCGAAGTTAACTCTAAAGCTTT
CGAAAAGAAATATGTATTTATTgtgagtttatctatagttattcaaacttattatttttatcagataacattttattaaaa
attttaaaatcagaagtcagcctgaagcagaagcagaagaagaagccaaactgctttttgaaatcttaacttattaaaagt
tagaattttccagGTTACAGCAAGCCAACTCTGCACGCTCTATCTTTTCAATGAGCAGGACTCGTTCACAATTGAACAAAT
TTCCAAAGCTATTGAAATGACTGCAAAGTCAACTTCGGCTATCGTAGGATCTCTTAATCCG
