# Supplementary material for: Punctuated Loci on Chromosome IV Determine Natural Variation in Orsay Virus Susceptibility of Caenorhabditis elegans Strains Bristol N2 and Hawaiian CB4856
Source: J Virol. 2021 May 24;95(12):e02430-20. doi: 10.1128/JVI.02430-20 (PMC8315983; doi:10.1128/JVI.02430-20)
Supplement: Supplementary file 5 [file jvi.02430-20-s0005.pdf]

Supplementary Table S4 Statistical comparisons between N2, CB4856, PHX1169 and PHX1170 – *An overview of the p-values obtained via t-tests to compare the parental and allele swap strains.*

**Strain A**	**Strain B**	**P-value t-test**
N2	CB4856	0.03417
N2	PHX1169	0.5076
N2	PHX1170	0.4107
CB4856	PHX1169	0.3587
CB4856	PHX1170	0.3054
